# Type-1 angiotensin receptor signaling in central nervous system myeloid cells is pathogenic during fatal alphavirus encephalitis in mice

**DOI:** 10.1186/s12974-016-0683-7

**Published:** 2016-08-25

**Authors:** Pennelope K. Blakely, Amanda K. Huber, David N. Irani

**Affiliations:** Holtom-Garrett Program in Neuroimmunology, Department of Neurology, University of Michigan Medical School, Room 4007, A. Alfred Taubman Biomedical Sciences Research Building, 109 Zina Pitcher Place, Ann Arbor, MI 48109-2200 USA

**Keywords:** Angiotensin II, Type-1 angiotensin II receptors, Angiotensin receptor blockers, Alphaviruses, Viral encephalitis, Nox, Oxidative injury

## Abstract

**Background:**

Alphaviruses can cause fatal encephalitis in humans. Natural infections occur via the bite of infected mosquitos, but aerosol transmissibility makes some of these viruses potential bioterrorism agents. Central nervous system (CNS) host responses contribute to alphavirus pathogenesis in experimental models and are logical therapeutic targets. We investigated whether reactive oxygen species (ROS) generated by nicotinamide adenine dinucleotide phosphate (NADPH) oxidase (Nox) activity within the CNS contributes to fatal alphavirus encephalitis in mice.

**Methods:**

Infected animals were treated systemically with the angiotensin receptor-blocking drug, telmisartan, given its ability to cross the blood-brain barrier, selectively block type-1 angiotensin receptors (AT1R), and inhibit Nox-derived ROS production in vascular smooth muscle and other extraneural tissues. Clinical, virological, biochemical, and histopathological outcomes were followed over time.

**Results:**

The importance of the angiotensin II (Ang II)/AT1R axis in disease pathogenesis was confirmed by demonstrating increased Ang II levels in the CNS following infection, enhanced disease survival when CNS Ang II production was suppressed, increased AT1R expression on microglia and tissue-infiltrating myeloid cells, and enhanced disease survival in AT1R-deficient mice compared to wild-type (WT) controls. Systemic administration of telmisartan protected WT mice from lethal encephalitis caused by two different alphaviruses in a dose-dependent manner without altering virus replication or exerting any anti-inflammatory effects in the CNS. Infection triggered up-regulation of multiple Nox subunits in the CNS, while drug treatment inhibited local Nox activity, ROS production, and oxidative neuronal damage. Telmisartan proved ineffective in Nox-deficient mice, demonstrating that this enzyme is its main target in this experimental setting.

**Conclusions:**

Nox-derived ROS, likely arising from CNS myeloid cells triggered by AT1R signaling, are pathogenic during fatal alphavirus encephalitis in mice. Systemically administered telmisartan at non-hypotensive doses targets Nox activity in the CNS to exert a neuroprotective effect. Disruption of this pathway may have broader implications for the treatment of related infections as well as for other CNS diseases driven by oxidative injury.

**Electronic supplementary material:**

The online version of this article (doi:10.1186/s12974-016-0683-7) contains supplementary material, which is available to authorized users.

## Background

Alphaviruses cause acute and sometimes fatal encephalomyelitis in humans. Most infections are spread via infected mosquito vectors, although some can be transmitted as aerosols making them potential bioterrorism agents [1]. The intentional release of an alphavirus amidst a large population center is a public health concern because antiviral agents effective against these pathogens are not available. One alphavirus, neuroadapted Sindbis virus (NSV), causes fatal encephalomyelitis in mice and closely reproduces many features of neurotropic alphavirus infections in humans [2, 3]. Like other alphaviruses, NSV targets neurons of the brain and spinal cord without causing direct infection of glial cells [3, 4]. The fate of neurons then determines disease outcome [5]. Not only does NSV cause direct virus-induced neuronal cell death [5], but substantial bystander injury to uninfected neurons also occurs [6, 7]. This bystander injury suggests that host responses contribute to alphavirus pathogenesis [8, 9], and recent investigations show that therapies targeting innate immune responses in the central nervous system (CNS) can protect infected hosts without altering virus tropism, replication, or clearance [7, 10, 11]. Microglia and astrocytes are both implicated in this bystander injury [11–14], but the molecular pathways through which innate host responses lead to neuronal injury during CNS alphavirus infections remain incompletely understood.

Among the numerous host responses known to promote neuronal injury in the CNS are reactive oxygen species (ROS) generated by activation of the nicotinamide adenine dinucleotide phosphate (NADPH) oxidase (Nox) enzyme complex [15, 16]. Indeed, aberrant activation of Nox in microglial cells is now implicated in a variety of neurodegenerative disease models [17–20]. The contributions of Nox-derived ROS generated as part of the host response to CNS viral infection are less clear; on one hand, these ROS may directly damage virus particles or lower the permissiveness of cells for virus replication, while on the other, their generation as a part of the respiratory burst by innate immune cells can produce significant collateral tissue damage [21]. A growing body of histochemical data show that oxidative neuronal injury occurs with CNS viral infections [21], suggesting that blockade of Nox activation could have therapeutic benefits in these diseases.

The vasoconstricting effects of angiotensin II (Ang II) on peripheral blood vessels occur via Nox-mediated production of superoxide anions by vascular smooth muscle cells [22, 23]. As a result, selective non-peptide antagonists of type-1 angiotensin II receptors (AT1R) that potently inhibit Nox activation have been developed as anti-hypertensive therapies for humans. More recently, several angiotensin II receptor blockers (ARB) have been shown to exert neuroprotective effects in animal disease models [24–27]. One ARB, telmisartan, is highly blood-brain barrier (BBB) penetrant and produces near complete and sustained blockade of AT1R without affecting type-2 angiotensin II receptor (AT2R) signaling [28]. Given the expanding use of ARB to suppress Nox-mediated ROS production in other non-vascular tissues [29], we considered telmisartan a useful reagent to test the hypothesis that Nox-mediated ROS production within the CNS is an important pathogenic event during experimental alphavirus encephalitis in mice. Our data show that systemically administered telmisartan blocks CNS Nox activity and ROS production induced by NSV infection, protects infected hosts, and suppresses oxidative neuronal damage. These findings suggest a broader potential applicability of ARB to target Nox activity in the setting of infectious, inflammatory, and even degenerative disorders of the human CNS.

## Methods

### Animals

Inbred C57BL/6 mice, mice heterozygous for deficiencies of the individual Nox subunits, p47^phox^ or gp91^phox^, both bred on a C57BL/6 background, mice expressing green fluorescent protein (GFP) in one allele of the CX3C chemokine receptor-1 (CX3CR1 or fractalkine receptor) bred on a C57BL/6 background (hereafter referred to as CX3CR1^GFP/+^ mice) and mice deficient in AT1R bred on a C57BL/6 background (hereafter referred to as AT1R knockout (KO) mice) were all obtained from Jackson Laboratories (Bar Harbor, ME). Double KO gp91^phox^/p47^phox^ mice (hereafter referred to as gp91/p47 double knockout (DKO) mice) were generated de novo and bred on site. Animals were housed under specific pathogen-free, barrier facility conditions on a 10-h/14-h light/dark cycle with food and water available ad libitum. All animal procedures were completed using isoflurane anesthesia (Abbott Laboratories, Chicago, IL). All animal experiments, including those incorporating paralysis or death as study endpoints, were conducted in strict accordance with protocols approved by the University of Michigan Committee on Use and Care of Animals as well as with federal guidelines.

### Induction of NSV encephalomyelitis and NSV-related animal manipulations

NSV viral stocks were grown and assayed for plaque formation on BHK-21 cells. Stock titers of 10^7^ plaque-forming units (pfu)/ml were stored at −80 °C until use. To induce encephalomyelitis, mice were injected with 1000 pfu of NSV or NSV-GFP suspended in 20 μl of phosphate-buffered saline (PBS) directly into the right cerebral hemisphere. For those experiments where tissue samples were not being collected for ex vivo analyses, each infected animal was scored by a blinded examiner into one of the following groups: (1) normal or minimally affected, (2) mild paralysis (some weakness of one or both hind limbs), (3) moderate paralysis (weakness of one hind limb, paralysis of the other hind limb), (4) severe paralysis (complete paralysis of both hind limbs), or (5) moribund/dead. Moribund mice were euthanized immediately and were considered to have died the following day for all statistical comparisons, as prior studies demonstrated that all animals reaching this disease stage never survived more than another 24 h [7, 10, 13]. Animals also received intraperitoneal (i.p.) injections of telmisartan (Sigma-Aldrich, St. Louis, MO) or a vehicle control twice daily in a volume of 200 μl of 0.9 % sodium chloride solution, pH 9.0. Such an alkalinized diluent was both necessary for and capable of maintaining full drug solubility. Doses up to 100 mg/kg/day were administered starting 12 h after viral challenge. Other mice received twice daily i.p. injections of captopril (10 mg/kg/day, Sigma-Aldrich) suspended in 200 μl of PBS or a PBS vehicle control. All of these experiments were conducted in strict accordance with protocol # PRO00005049 approved by the University of Michigan Committee on Use and Care of Animals.

At predetermined disease stages, some mice were euthanized via transcardiac perfusion with PBS under anesthesia. For animals where in vivo labeling of ROS production was desired, mice were first injected i.p. with 50 mg/kg dihydroethidium (DHE, Sigma-Aldrich) 1 h prior to perfusion. For all tissue enzyme immunoassays (EIA), bead-based multi-analyte cytokine and chemokine assays, Western blots, flow cytometry-based sorting of immune cell subsets, virus titration assays, and biochemical assays of tissue superoxide production, brains and spinal cords were quickly dissected for further processing. For immunohistochemical and immunofluorescence studies, animals were perfused a second time with chilled PBS containing 4 % paraformaldehyde (PFA) before dissection.

### Induction of western equine encephalitis virus encephalitis

The Cba 87 strain of western equine encephalitis virus (WEEV) was generated from the complementary DNA (cDNA) clone pWE2000 as previously described [30, 31]. A low passage stock of infectious virus generated in Vero cells was expanded twice in C6/36 mosquito cells to produce viral stocks containing 10^7^ pfu/ml. All virus aliquots were stored at −80 °C until use. Experiments using infectious WEEV were conducted in an animal-compatible, federally certified biosafety level 3 (BSL3) containment facility in strict accordance with standard operating procedures and protocols approved by both the University of Michigan Institutional Biosafety Committee and the University of Michigan Committee on Use and Care of Animals (animal protocol # PRO00005204). Mice were housed in our animal BSL3 facility on a 10-h/14-h light/dark cycle with food and water available ad libitum.

For WEEV infections, recipient mice were inoculated subcutaneously with 10^4^ pfu of Cba 87 suspended in a total volume of 50 μl of PBS. Cohorts of infected mice were weighed and scored daily using a clinical rating scale previously established for WEEV infection: 0, normal; 1, slightly ruffled fur, no visible signs of infection; 2, very ruffled fur, definite signs of infection with reduced cage activity; 3, very ruffled fur, hunched posture, reduced mobility; 4, very ruffled fur, hunched posture, little to no mobility, rapid breathing (moribund); and 5, dead [32]. All animals also received i.p. injections of telmisartan (Sigma-Aldrich) or a vehicle control twice daily in a total volume of 200 μl of 0.9 % sodium chloride solution, pH 9.0, at a dose of 100 mg/kg/day starting 12 h after viral challenge. Animals reaching a disease score of 4 were euthanized immediately and were considered to have died the following day for all statistical comparisons, as prior disease progression studies demonstrated that moribund mice never survived more than an additional 24 h [32].

### Measurement of Ang II levels and cytokine and chemokine concentrations in tissue extracts

Control and NSV-infected mice were perfused with PBS, and brains and spinal cords extracted, snap-frozen on dry ice, and stored at −80 °C until use. Thawed tissues were minced and homogenized in 0.5 ml of PBS containing a protease inhibitor cocktail (Roche Life Science, Indianapolis, IN). Homogenates were centrifuged to pellet all remaining tissue debris. The total protein content in each tissue supernatant was measured using the Pierce Coomassie Protein Assay Reagent (Thermo Fisher Scientific, Rockford, IL). Ang II levels were measured in each sample using a commercially available EIA kit (Cayman Chemical Company, Ann Arbor, MI). The results presented reflect pg Ang II/mg of total protein extracted from tissue of four to six animals at each time point examined. The Milliplex mouse cytokine detection system (EMD Millipore, Billerica, MA) was used according to the manufacturer’s instructions to quantify cytokine and chemokine levels directly in tissue extracts. Plates were read on a Luminex 200 instrument (EMD Millipore), and cytokine and chemokine concentrations (pg/ml) calculated by the BioPlex manager software (Bio-Rad, Hercules, CA) using standard curves. Results presented reflect the mean ± standard error of the mean (SEM) of cytokine or chemokine quantities per milliliter of plasma or per milligram of total extracted tissue protein from five animals at each time point. The lower limit of detection for these assays was 1.6 pg/ml.

### Tissue Western blots

Brains and spinal cords were homogenized in a tissue lysis buffer (10 mM Tris, 1 % sodium dodecyl sulfate (SDS), 1 mM sodium orthovandate, pH 7.6) supplemented with a commercial protease inhibitor cocktail (Roche Life Science). Lysates were centrifuged to remove undigested tissue debris and the total protein concentration of each supernatant was determined using the Pierce Coomassie Protein Assay Reagent (Thermo Fisher Scientific). Samples were boiled in 4× protein sample buffer and 20 μg/well run on SDS-polyacrylamide gels. Proteins were transferred to PVDF membranes and blocked overnight at 4 °C in a 5 % non-fat skim milk solution in Tris-buffered saline (TBS) containing 0.5 % Tween 20. Membranes were then incubated with one of the following primary antibodies: polyclonal goat anti-AT1R (1:500, Santa Cruz Biotechnology, Santa Cruz, CA), polyclonal rabbit anti-gp91 (1:1000, Abcam, Cambridge, MA), or polyclonal rabbit anti-p47 (1:500, EMD Millipore) for 1 h at room temperature. All primary antibodies were raised against synthetic peptides derived from the corresponding human gene sequence, and both cross-reactivity and specificity with the corresponding mouse protein previously established [33–35]. Following five washes, membranes were then incubated with either rabbit anti-goat horseradish peroxidase (HRP)-conjugated secondary antibody or goat anti-rabbit HRP-conjugated secondary antibody (both used at 1:10,000, EMD Millipore) for 1 h at room temperature. Membranes were washed five times again, and the HRP signal detected using ECL Western blotting detection reagent (GE Healthcare Life Sciences, Pittsburgh, PA) on X-ray film. Membranes were then stripped using Western blot stripping buffer (Thermo Fisher Scientific) and relabeled with β-actin loading control antibody (1:5000, Thermo Fisher Scientific) using the same steps described above. Once all the β-actin signals were obtained, all band densities were quantified using the ImageJ software package (NIH, Bethesda, MD). The band density for each protein was first normalized to the β-actin signal detected in the same lane, and the mean of the uninfected samples were then set to an arbitrary expression level of 1.0. Relative protein expression across the full course of acute NSV infection was determined compared to uninfected controls, and relative expression in five samples at each disease stage was analyzed for statistical significance.

### Tissue staining and imaging procedures

Unless otherwise specified, all tissues used for histological studies were post-fixed for 6 h in 4 % PFA in PBS, cryopreserved overnight in 30 % sucrose in PBS, and snap-frozen in CRYO-OCT Compound (Thermo Fisher Scientific). Eight micron frozen sections were cut, collected on SuperFrost Plus slides (Thermo Fisher Scientific) and stored at −20 °C until staining.

#### Fluorescence

Sections of NSV-GFP-infected tissues prelabeled with DHE were imaged without further manipulation. Other sections were brought to room temperature, washed in PBS, and boiled for 20 min in 0.01 M citric acid in PBS (pH 6.0) to unmask tissue antigens. Tissue sections were then permeabilized for 5 min in 0.1 % Triton X-100 in PBS and blocked for 30 min in 5 % normal goat serum (NGS). Sections from NSV-infected CX3CR1^GFP/+^ mice were incubated for 1 h at room temperature with polyclonal rabbit anti-AT1R (1:100, Santa Cruz Biotechnology), washed three times in PBS, incubated with rhodamine-conjugated goat anti-rabbit secondary antibody (1:200, eBioscience, San Diego, CA) for 1 h at room temperature, and washed again prior to coverslipping with Fluoromount G (eBiosciences) and imaging. Neuronal damage in the brain was assessed in sections prepared through the hippocampal formations of naïve and NSV-infected C57BL/6 mice. Before staining, each section was incubated in 0.1 % Triton X-100 for 15 min to expose intracellular antigens. Slides were then stained in 0.0001 % Fluoro-Jade C compound (EMD Millipore) in 1 % acetic acid for 10 min. After further washing, slides were dried, counterstained with hematoxylin, dehydrated in xylene, and coverslipped using VectaMount permanent mounting media (Vector Laboratories, Burlingame, CA). Fluorescence was imaged using a Nikon Ti-U inverted microscope equipped with a CoolSNAP EZ CCD digital camera (Photometrics, Tucson, AZ) supported by the NIS-Elements Basic Research acquisition and analysis software package (Nikon Instruments Inc., Melville, NY). To quantify neuronal damage, Fluoro-Jade C-positive cells (degenerating neurons) and hematoxylin-positive cells (all neurons) were counted on duplicate slides from each hippocampus of triplicate mice for each experimental condition to calculate the proportion of Fluoro-Jade-positive neurons.

#### Immunoperoxidase staining

For immunoperoxidase staining, permeabilized sections were first treated with 1 % hydrogen peroxide in methanol to block endogenous tissue peroxidase, and then blocked in 2 % NGS. Slides were washed, incubated with polyclonal rabbit anti-4-hydroxynonenal (4-HNE, 1:250, Abcam) for 1 h at room temperature, washed again, and then treated with biotin-labeled goat anti-rabbit IgG (Vector Laboratories) at a 1:200 dilution for another hour at room temperature. These steps were followed by sequential incubations with avidin-DH-biotin complex (Vector Laboratories) and then 0.5 mg/ml 3,3′-diaminobenzidine (Sigma-Aldrich) in PBS containing 0.01 % hydrogen peroxide. All slides were counterstained with hematoxylin and mounted with coverslips using Permount mounting medium (Thermo Fisher Scientific) for light microscopy. Slides were imaged using a Nikon Ti-U inverted microscope equipped with a Nikon DS-Fi-1 digital camera and supported by the NIS-Elements Basic Research acquisition and analysis software package (Nikon Instruments Inc.).

#### Silver staining

Neuronal damage in the spinal cord during NSV infection was assessed by quantifying the axonal processes of lumbar ventral spinal nerve roots as these all originate from motor neurons in the lumbar spinal cord that innervate the hind limb musculature. These assays were conducted according to our published methods [36]. In brief, sections of the entire lumbar spinal column at the L4–L5 level were first decalcified (Immunocal, Decal Corporation, Tallman, NY) and embedded in paraffin. Sections were then stained using a modified Bielchowsky silver staining method to label neurofilament proteins in each nerve axon, as described [36]. Slides were imaged using a Nikon Ti-U inverted microscope equipped with a Nikon DS-Fi-1 digital camera and supported by the NIS-Elements Basic Research acquisition and analysis software package (Nikon Instruments Inc.). Axonal density (the number of intact axons per cross-sectional area of each nerve root) was determined for the right and left L4 and L5 ventral nerve roots from a minimum of four animals in each experimental group.

### Flow cytometry-based analysis and separation of CNS myeloid cell subsets

Six days after NSV challenge, ten anesthetized C57BL/6 mice underwent transcardiac perfusion with chilled PBS. A parallel cohort of five mice were infected and also treated with 100 mg/kg/day of telmisartan for 6 days followed by transcardiac perfusion. Brains and spinal cords were collected from each mouse and homogenized into small fragments. Tissue was suspended in Hank’s balanced salt solution (HBSS) containing 28 U/ml DNase (Sigma-Aldrich) for 30 min at 37 °C. Infiltrating mononuclear cells (MNC) were isolated from tissue digests of five telmisartan-treated and five untreated mice by centrifugation over 30 %/70 % Percoll gradients (GE Healthcare Life Sciences), counted, washed extensively with PBS containing 2 % fetal bovine serum (FBS), and stained with fluorescently conjugated anti-CD45, anti-CD11b, anti-Ly6C, anti-Ly6G, and anti-CD11c antibodies for myeloid cell phenotyping, as well as anti-CD40 and anti-CD86 to assess myeloid cell activation (all from eBiosciences). The following staining patterns defined each myeloid cell subset: CD45^low^/CD11b + (microglia), CD45^high^/CD11b+/CD11c-/Ly6C+/Ly6G- (macrophages), CD45^high^/CD11b+/CD11c-/Ly6C-/Ly6G- (monocytes), CD45^high^/CD11b+/CD11c-/Ly6C-/Ly6G+ (neutrophils), and CD45^high^/CD11b+/CD11c + (dendritic cells). In the remaining five NSV-infected mice, CNS mononuclear cells were pooled and the endogenous and recruited myeloid cell subsets were physically separated from one another into CD45^low^/CD11b + (microglia) and CD45^high^/CD11b + (all infiltrating myeloid cells) populations using a BD FACSAria high-speed cell sorter (BD Biosciences, San Jose, CA). Cells were stored in PrepProtect RNA stabilization solution (Miltenyi Biotec, Auburn, CA) at −20 °C until RNA isolation could be performed.

### Quantitative PCR determination of *agtr1a* expression in CNS myeloid cell subsets

Flow sorted cell subsets were thawed, pelleted, and carefully removed from the PrepProtect solution. Total RNA was isolated from each cell population and cDNA generated using a high-capacity cDNA reverse transcription kit according to the manufacturer’s instructions (Thermo Fisher Scientific). Quantitative PCR (qPCR) was performed to measure *agtr1a* and *β-actin* mRNA transcripts using the MyiQ Single Color Real-Time PCR Detection System and a Bio-Rad iQ5 cycler (Bio-Rad, Hercules, CA). TaqMan® gene expression assays for both *agtr1a* and *β-actin* were obtained from Thermo Fisher Scientific. Levels of *agtr1a* transcripts were calculated relative to *β-actin* using the following formula: 2^[Ct (β-actin) − Ct (target gene)] × 1000, where Ct is the threshold cycle at which the fluorescent signal became significantly higher than background. Results presented reflect relative *agtr1a* mRNA expression in each cell population done in three experimental replicates.

### Tissue viral titrations

To measure the amount of infectious virus present in CNS tissues, animals were perfused extensively with chilled PBS and brains and spinal cords were extracted, weighed, snap-frozen on dry ice, and stored at −80 °C until virus titrations assays were performed. At that time, 20 % (*w*/*v*) homogenates of each sample were prepared in Dulbecco’s modified Eagle’s medium (DMEM, Sigma-Aldrich), and serial tenfold dilutions of each homogenate were assayed for plaque formation on monolayers of BHK-21 cells. Results presented are the mean ± SEM of the log_10_ of viral pfu per gram of tissue derived from a minimum of three animals at each time point.

### Measurement of tissue superoxide-generating activity

The capacity of CNS tissues to generate ROS was measured directly ex vivo using the well-established cytochrome c reduction assay on fresh tissue membrane extracts, as described [37, 38]. Briefly, animals were perfused with chilled PBS, and brains and spinal cords were extracted, weighed, and homogenized to a final concentration of 1 mg/ml total tissue protein in DMEM. One hundred-eighty microliters of each homogenate was then added to 96-well, flat-bottom plates in duplicate. Purified superoxide dismutase (SOD, Sigma-Aldrich) was then added to half the wells at a final concentration of 200 U/ml, while DMEM was added to the remaining wells as a control. All wells then received 500 μmol/l purified cytochrome c (Sigma-Aldrich) and 100 μmol/l purified NADPH (Sigma-Aldrich) as a specific electron donor, and plates were incubated at 37 °C for 30 min. Immediately thereafter, the absorbance of each well was read at 540, 550, and 560 nm using a PowerWave HT spectrophotometer (BioTek, Winooski, VT). Tissue O_2_^−^ production by each sample was calculated by first subtracting the average optical density (OD) readings at 540 and 560 nm from the average OD reading at 550 nm (ΔOD). The difference in ΔOD value in the absence and presence of SOD was then divided by the extinction coefficient of cytochrome c using the following formula: (ΔOD without SOD – ΔOD with SOD)/21.1. Results are presented as nmol O_2_^−^ produced/mg tissue protein.

### Statistical comparisons

The Prism 5.0 software package (GraphPad Software, La Jolla, CA) was used for all statistical analyses. Student’s *t* test was applied when comparing a single group under two experimental conditions, a one-way analysis of variance (ANOVA) with a post hoc Bonferroni’s multiple comparison test was used to investigate the significance of a single group’s change over time, while a two-way ANOVA with a post hoc Bonferroni’s multiple comparison test was utilized to compare experimental findings between two groups over time. Differences in outcome among individual cohorts of infected mice were determined using a log-rank (Mantel-Cox) test. In all cases, differences at a *p* < 0.05 level were considered significant.

## Results

### Role of the Ang II-AT1R axis in the CNS during NSV encephalomyelitis

**Fig. 1:**
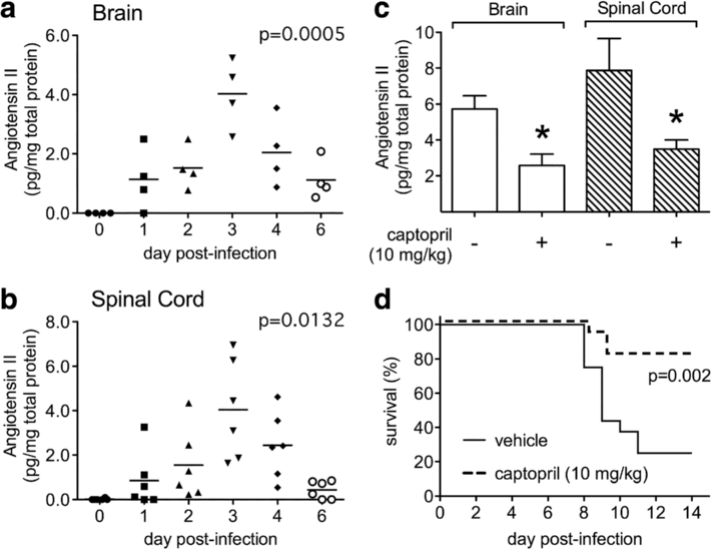
Ang II levels increase in both the brains (**a**) and spinal cords (**b**) of mice with NSV encephalomyelitis, while treatment of NSV-infected mice with the ACE inhibitor, captopril, suppresses Ang II induction in the CNS (**c**) and enhances disease survival (**d**). Tissue Ang II levels were measured by EIA in homogenates collected from uninfected (day 0) or NSV-infected animals and normalized to the total extracted protein content of each sample. Values measured in individual mice (*n* = 4–6 samples per time point) and mean concentrations at each time point are shown. One-way ANOVA confirmed that Ang II levels change significantly in both CNS tissue compartments over time (*p* = 0.0005 for brain, *p* = 0.0132 for spinal cord). Treatment of mice with captopril (10 mg/kg/day) suppressed Ang II induction on day 3 post-infection compared to mice given a vehicle control (*n* = 5 samples per group, *p < 0.05 by Student’s *t* test). This same treatment regimen enhanced overall disease survival compared to mice given a vehicle control (*n* = 7 mice per group, *p* = 0.002 using a log-rank (Mantel-Cox) test)

To explore whether Ang II-AT1R signaling influences disease outcome in mice with NSV encephalomyelitis, we measured Ang II concentrations directly in CNS tissues over time. These assays showed that Ang II levels increased in both the brains and spinal cords of mice over the first few days following NSV infection (Fig. 1a, b). Furthermore, local Ang II production was suppressed via systemic administration of captopril (Fig. 1c), an angiotensin converting enzyme (ACE) inhibitor that prevents Ang II cleavage from its precursor, angiotensinogen (AGT). Importantly, mice treated with this drug showed enhanced survival compared to animals receiving a vehicle control (Fig. 1d). These data suggest that local Ang II production could be an important early event that triggers CNS damage and leads to fatal disease in mice with NSV infection.

**Fig. 2:**
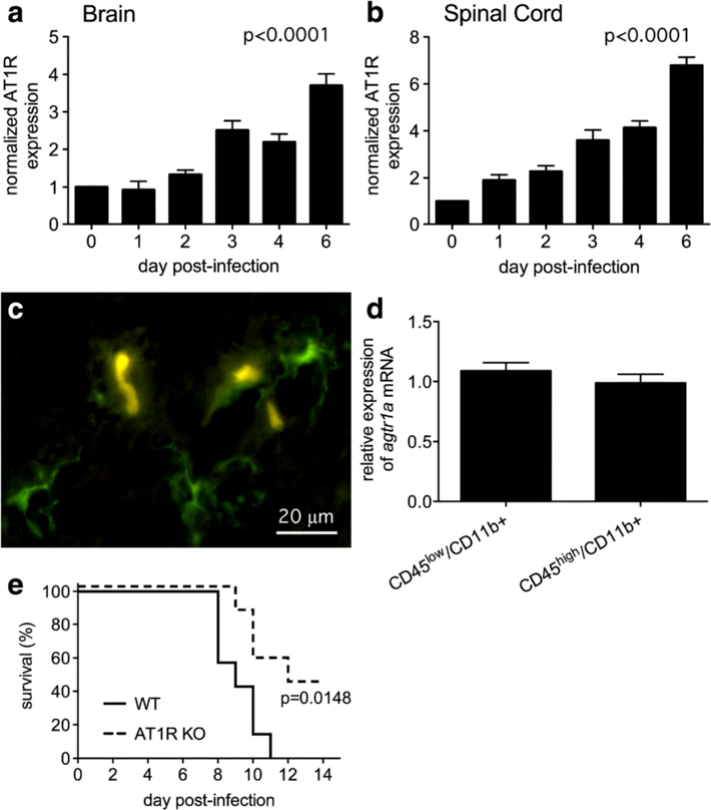
AT1R levels progressively increase in both the brains (**a**) and spinal cords (**b**) of mice with NSV encephalomyelitis; receptor expression remains restricted to microglia (**c**) and infiltrating monocytes (**d**) in infected mice and receptor deletion confers significant protection against lethal disease (**e**). Normalized AT1R expression in whole tissue extracts (*n* = 5 samples per time point) was determined by Western blot. One-way ANOVA confirmed that AT1R levels changed significantly in both CNS compartments over time (*p* < 0.0001 for both brain and spinal cord). Using transgenic mice selectively expressing GFP in CX3CR1+ cells, AT1R expression was found on a subset of microglia from the hippocampal formations of uninfected animals (**c**). AT1R staining was not detected on any CX3CR1− cells. *Bar* = 20 μm. At peak inflammation (day 6), CD45^low^/CD11b + microglia and CD45^high^/CD11b + infiltrating monocytes expressed equivalent levels of *agtr1a* mRNA (*n* = 5 samples per group) (**d**). Finally, global AT1R KO mice survived NSV infection more readily than WT controls (*n* = 7 mice per group, *p* = 0.0148 using a log-rank (Mantel-Cox) test) (**e**)

We next characterized CNS AT1R expression, demonstrating that tissue receptor levels increased steadily in both the brains and spinal cords of NSV-infected animals (Fig. 2a, b). A representative Western blot is shown (Additional file 1: Figure S1). Using CX3CR1^GFP/+^ mice with constitutively green microglia [39], AT1R expression was identified by histochemical staining on a subset of these cells in the brains of naïve animals (Fig. 2c). Because CX3CR1-positive cells could also represent GFP-positive monocytes infiltrating from the periphery [39], these two cell populations were separated from the brains of NSV-infected animals by flow sorting for ex vivo analysis. Using this approach, anti-AT1R antibodies proved unreliable to detect surface expression by flow cytometry, but *agtr1a* transcripts were found at equivalent levels in CD45^low^/CD11b + microglia and CD45^high^/CD11b + infiltrating myeloid cells at peak disease (Fig. 2d). No AT1R expression was detected by histochemical staining on CX3CR1-negative cells in either control or NSV-infected tissues (Fig. 2c and data not shown). Finally, AT1R KO mice challenged with NSV demonstrated improved survival compared to wild-type (WT) controls (Fig. 2e). These results confirm that AT1R signaling contributes to NSV pathogenesis. Furthermore, local pharmacological blockade of AT1R in the CNS, if achieved, would act mainly on the endogenous and recruited myeloid cell populations in this disease setting.

### Effects of a systemically administered AT1R antagonist on the course of lethal alphavirus encephalomyelitis in mice

**Fig. 3:**
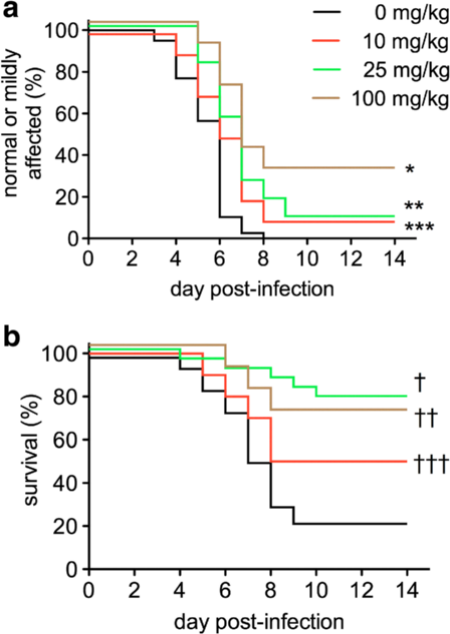
Telmisartan protects mice against both moderate or severe hind limb paralysis (**a**) and death (**b**) following NSV challenge in a dose-dependent manner. Cohorts of animals (*n* = 15–23 mice per group) were inoculated with NSV and treated twice daily with either telmisartan or a vehicle control at the daily doses indicated. Blinded examiners scored each animal daily as described. The proportion of mice in each group that remained either normal/mildly affected (**a**) or alive (**b**) was calculated over time. Statistical differences between drug-treated groups and vehicle-treated controls were determined using a log-rank (Mantel-Cox) test. Individual *p* values are as follows: *p < 0.0001; **p = 0.0002; ***p = 0.0244; †*p* < 0.0001; ††*p* = 0.0148; †††*p* = 0.1514

We next investigated the effects of telmisartan on the course of NSV encephalomyelitis, a drug known to effectively penetrate the BBB and cause sustained AT1R blockade without affecting AT2R signaling [28]. Cohorts of mice were treated twice daily with the drug beginning 12 h after viral challenge. Escalating doses up to 100 mg/kg/day were used, below those previously shown to lower systemic arterial blood pressure in rodents [40]. Analyses of disease outcomes in these cohorts showed that drug treatment attenuated the development of severe hind limb paralysis and prevented death in a dose-dependent manner compared to animals given a vehicle control (Fig. 3). These data further support a role for AT1R signaling in NSV pathogenesis.

**Fig. 4:**
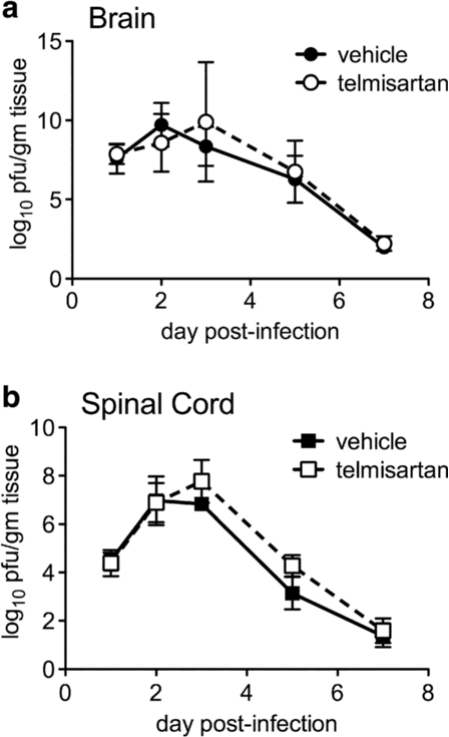
Telmisartan has no effect on the replication or clearance of NSV from the brains (**a**) or spinal cords (**b**) of NSV-infected animals. Mice were inoculated with NSV and treated with telmisartan (100 mg/kg/day) or a vehicle control. Replicate mice were sacrificed at predetermined intervals, and viral titers were measured in each tissue sample by plaque titration assay. Mean ± SEM of the log_10_ of tissue plaque-forming units (pfu) per gram of tissue are shown. Two-way ANOVA confirmed that the virus replication curves from vehicle- and telmisartan-treated mice are not different from each other (*p* = 0.74 for brain, *p* = 0.31 for spinal cord)

Virus titrations were performed on tissues derived from vehicle- and telmisartan-treated animals to investigate whether the drug had any effect on CNS virus replication or spread. These assays showed that virus replication was completely unaltered in both the brains and spinal cords of NSV-infected mice treated with the highest protective dose of telmisartan examined (Fig. 4). Furthermore, infection with NSV-GFP demonstrated that drug treatment did not change virus tropism for neurons or reduce the number of infected cells in heavily targeted CNS regions such as the hippocampus or the lumbar spinal cord (data not shown). These results show that telmisartan produces benefits in mice with NSV encephalomyelitis through a mechanism other than one involving direct antiviral activity.

**Fig. 5:**
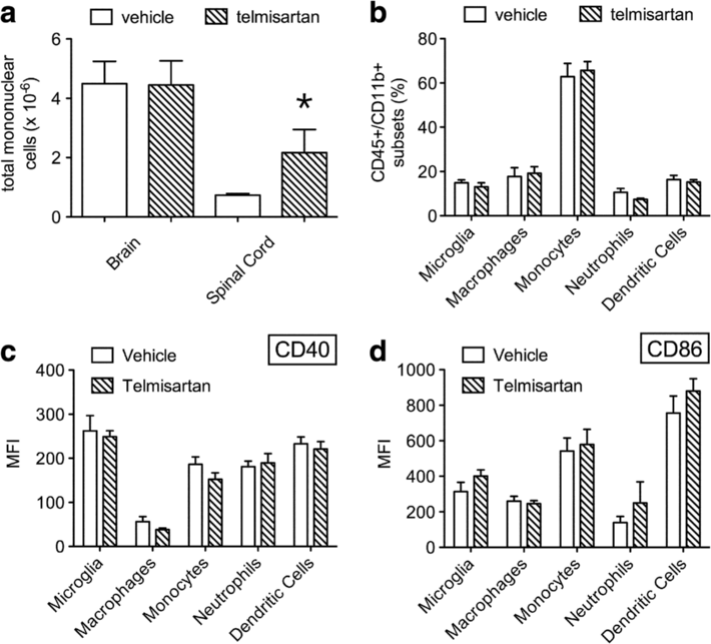
Telmisartan has no effect on the local CNS inflammatory response induced following NSV infection. At peak inflammation (day 6), total MNC infiltration was not suppressed in either the brains or spinal cords of mice (*n* = 5 mice per group) treated with telmisartan (100 mg/kg/day) compared to a vehicle control (**a**). By flow cytometry, the proportions of different brain-infiltrating myeloid cell subsets (CD45+/CD11b+) were not different between these two groups (**b**). Likewise, expression of CD40 (**c**) and CD86 (**d**) were not changed on any resident or CNS-infiltrating myeloid cell subset in the setting of drug treatment as assessed by the mean fluorescence intensity (MFI) of staining for the two activation markers

**Fig. 6:**
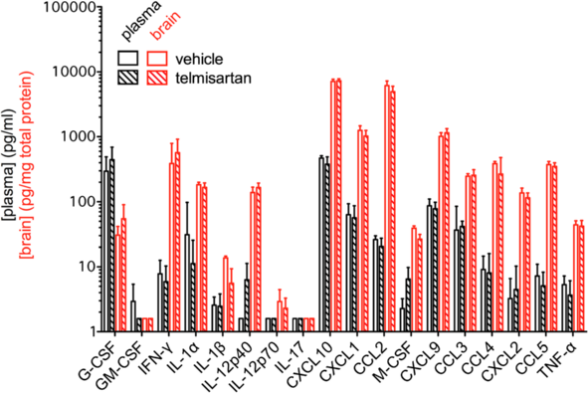
Telmisartan has no effect on the systemic or local CNS production of myeloid-related immune factors following NSV challenge. At peak inflammation (day 6), plasma and brain tissue was collected from mice (*n* = 5 mice per group) treated with telmisartan (100 mg/kg/day) compared to a vehicle control. Plasma samples were assayed without further manipulation. Tissue homogenates were first normalized to the total extracted protein content of each sample. Plasma and brain levels of myeloid-related immune factors were measured using a commercial assay according to the manufacturer’s instructions. Plasma (pg/ml) or brain (pg/mg total tissue protein) concentrations are shown. No differences in any vehicle- versus telmisartan-treated brain concentrations were identified

To investigate whether telmisartan exerted any anti-inflammatory effects in NSV-infected animals, we measured total MNC infiltration into the CNS, the recruitment of individual myeloid cell subsets into the brain, the cellular activation status of these CNS myeloid cells, and circulating and CNS levels of myeloid cell-related immune factors after 6 days of treatment. Ex vivo analyses of CNS isolates from infected animals showed that total tissue MNC were not reduced in telmisartan- compared to vehicle-treated mice (Fig. 5a). The proportions of five distinct myeloid cell subsets were equivalent between the two groups (Fig. 5b), and telmisartan had no effect on expression levels of the activation markers, CD40 and CD86, on any of these cell types (Fig. 5c, d). When a panel of myeloid factors was measured in both plasma and brain tissue from NSV-infected animals, telmisartan had minimal effects on the production of any of these mediators in either tissue compartment (Fig. 6). We conclude that telmisartan does not exert a global anti-inflammatory effect in either the periphery or the CNS during NSV infection.

**Fig. 7:**
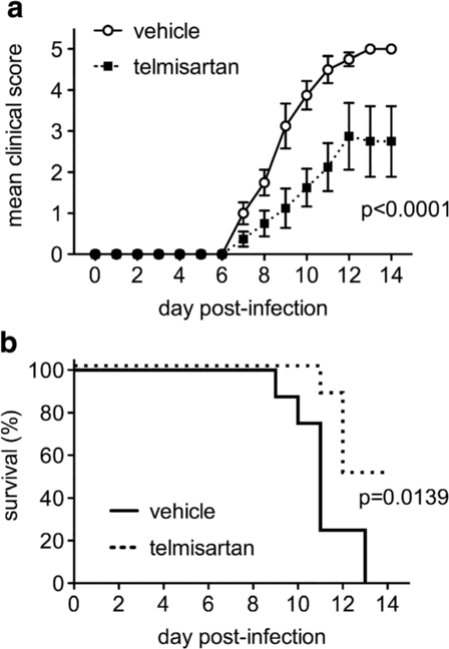
Mice treated with telmisartan developed milder disease (**a**) and were protected from death (**b**) following challenge with the human alphavirus, WEEV. Mice were inoculated with WEEV and treated with either telmisartan (100 mg/kg/day) or a vehicle control. Blinded examiners scored each animal daily for both disease severity and survival. The mean ± SEM disease score (**a**) and the proportion of animals surviving (**b**) are shown (*n* = 8 mice/group). A two-way ANOVA confirmed that drug treatment lessened disease severity (*p* < 0.0001), while a log-rank (Mantel-Cox) test confirmed that telmisartan enhanced disease survival (*p* = 0.0139)

To determine the broader relevance of telmisartan as a treatment for alphavirus encephalitis, separate cohorts of mice were challenged with the known human pathogen, WEEV. Much like what was observed in NSV-infected animals, systemic administration of telmisartan at a similar dose caused reduced disease severity in WEEV-infected mice compared to vehicle-treated controls (Fig. 7a). Half of the treated animals survived infection compared to what was uniformly lethal without the drug (Fig. 7b). These data confirm that telmisartan can protect a significant proportion of mice using a lethal challenge model of a clinically relevant alphavirus. As ARB drugs are in widespread human use, and as antiviral drugs effective against the alphaviruses still remain a long way from human application [41, 42], blockade of this signaling pathway might be considered for testing in both equine and human cases of acute alphavirus encephalitis.

### Effects of telmisartan on Nox activation, ROS production, and oxidative neuronal injury in the CNS during NSV encephalomyelitis

**Fig. 8:**
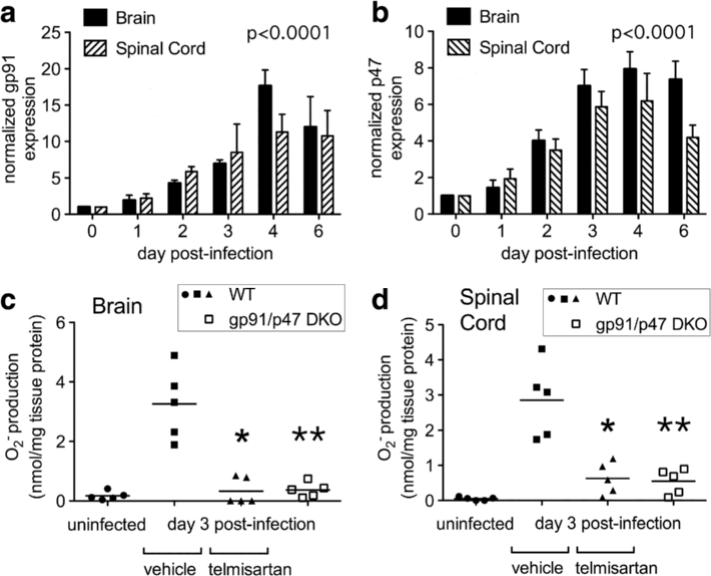
Nox subunits are upregulated in the CNS following NSV challenge (**a**, **b**), and enzymatic activity capable of generating ROS is both induced in the brains (**c**) and spinal cords (**d**) of infected animals and inhibited by telmisartan as well as by genetic deletion of Nox subunits. Normalized gp91 and p47 expression in whole tissue extracts was determined by Western blot as described in the “Methods” section. One-way ANOVA confirmed that levels of both Nox subunits changed significantly in both CNS regions over time (*p* < 0.0001). ROS-generating enzyme activity was measured directly in tissue extracts as described in the “Methods” section. Values derived from individual mice as well as mean enzyme activity levels are shown for each experimental condition (*n* = 5 mice/group). Student’s *t* test was used to analyze the degree to which telmisartan (100 mg/kg/day) or Nox subunit deletion suppressed enzyme activity in tissues derived from NSV-infected animals compared to those from vehicle-treated controls (*p = 0.0009 in brain and *p* = 0.0026 in spinal cord; **p = 0.0005 in brain and *p* = 0.0004 in spinal cord)

Given the known capacity of ARBs to suppress Nox-mediated ROS production in both vascular smooth muscle and renal podocytes [22, 23, 29], we next sought to determine whether telmisartan suppresses tissue ROS production and oxidative neuronal injury as a potential mechanism through which it protected NSV-infected animals. To investigate Nox activity in the CNS during disease, tissue subunit expression and tissue enzyme activity were measured ex vivo. Via Western blot, multiple Nox subunits were induced in both the brains and spinal cords of NSV-infected animals over time (Fig. 8a, b). A representative blot is shown (Additional file 2: Figure S2). Using an established method to quantify superoxide production in tissue extracts via ex vivo reduction of cytochrome c and exogenous NADPH as an electron donor [37, 38], we found clear biochemical evidence that enzymes capable of generating ROS were induced in both the brains and spinal cords of animals 72 h following NSV challenge (Fig. 8c, d). Furthermore, this enzyme activity was potently suppressed in tissues derived from infected mice treated with telmisartan, and it was ablated in samples collected from infected gp91/p47 DKO animals not treated with the drug (Fig. 8c, d). On the other hand, telmisartan had no effect on the induction of Nox subunits in the CNS by Western blot (data not shown). We conclude that Nox is the principal enzymatic source of tissue ROS in this disease setting, and that telmisartan suppresses Nox activity in the CNS without inhibiting expression of the enzyme complex itself.

**Fig. 9:**
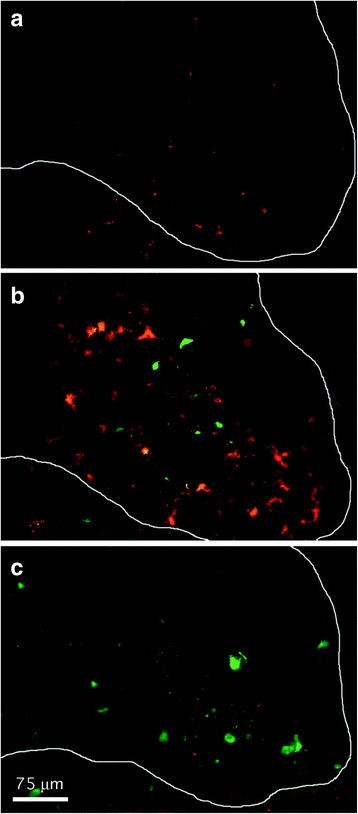
In vivo labeling shows that ROS are induced in ventral gray matter of the lumbar spinal cord (outlined) following NSV challenge and that ROS induction is suppressed in infected animals treated with telmisartan. ROS-containing cells (*red*) were labeled by injecting mice with DHE just prior to sacrifice as described in the “Methods” section. A GFP-expressing virus was used to identify NSV-infected neurons (*green*). Negligible ROS staining was observed in uninfected control animals (**a**). Four days after NSV challenge, ROS were detected in relative proximity to, but not necessarily within, infected neurons (**b**). Telmisartan administration (100 mg/kg/day) abolished nearly all ROS labeling in infected animals at this time point (**c**). Calibration *bar* = 75 μm for all panels

To investigate the spatial expression of ROS directly in CNS tissues, animals were infused with DHE immediately prior to sacrifice. This cell-permeable dye gets oxidized to the fluorescent compound, ethidium, by intracellular ROS and trapped within cells [43]. We found that DHE labeling was increased in the spinal cord 96 h after viral challenge when compared to labeled tissue derived from uninfected animals. This ROS signal remained largely confined to gray matter regions where infected cells were also found (Fig. 9a, b). Systemic treatment with telmisartan at protective doses largely abolished this DHE staining (Fig. 9c). Similar patterns of DHE labeling suppressed by telmisartan treatment were also observed in the brain, and no DHE labeling above background was found in tissues from NSV-infected but otherwise untreated gp91/p47 DKO mice (data not shown). Since drug treatment did not inhibit virus replication in either the brain or spinal cord (Fig. 4), we conclude that ROS do not exert any antiviral effect during alphavirus encephalitis.

**Fig. 10:**
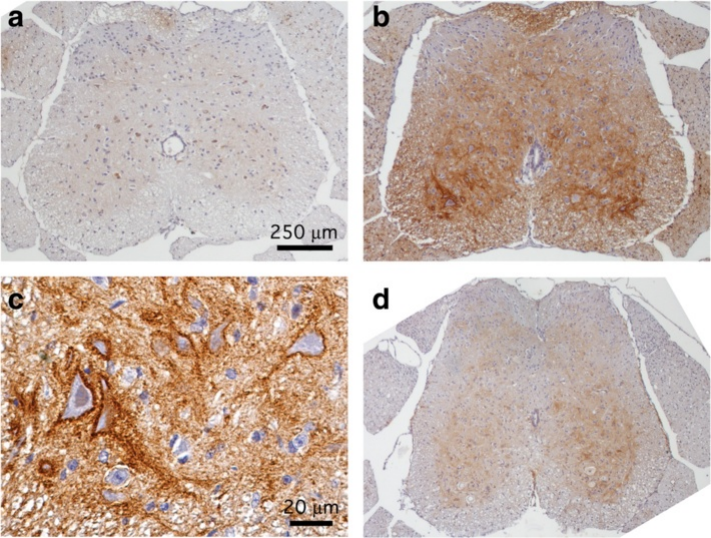
Telmisartan blocks oxidative changes to neurons in the spinal cords of NSV-infected mice. Immunohistochemical staining for 4-HNE, a lipid peroxidation product that accumulates in cell membranes following ROS exposure, is induced 4 days after NSV infection (**b**) compared to expression in uninfected control animals (**a**). Higher magnification views show prominent staining of neuronal cell membranes with less intense labeling of neuropil in ventral gray matter of infected animals (**c**). Treatment of NSV-infected mice with telmisartan (100 mg/kg/day) for 4 days blocks most of the observed 4-HNE immunoreactivity (**d**). Calibration *bar* = 250 μm in panels **a**, **b** and **d**; 20 μm in panel **c**

ROS generated as part of an inflammatory response can damage cellular lipids, proteins, and nucleic acids. A lipid peroxidation product detectable in cell membranes, 4-HNE, is both a validated marker for, as well as a direct mediator of, oxidative injury to neurons [44, 45]. We found that CNS tissues derived from NSV-infected animals showed prominent 4-HNE immunoreactivity in spinal gray matter within 72 h of viral challenge compared to uninfected controls (Fig. 10a, b). At higher magnification, heavy labeling of neuronal cell bodies, with moderate staining of gray matter neuropil, was observed (Fig. 10c). Treatment with telmisartan visibly reduced 4-HNE staining observed in infected tissue samples (Fig. 10d). Similar patterns of 4-HNE immunoreactivity were also identified in the hippocampus (data not shown).

**Fig. 11:**
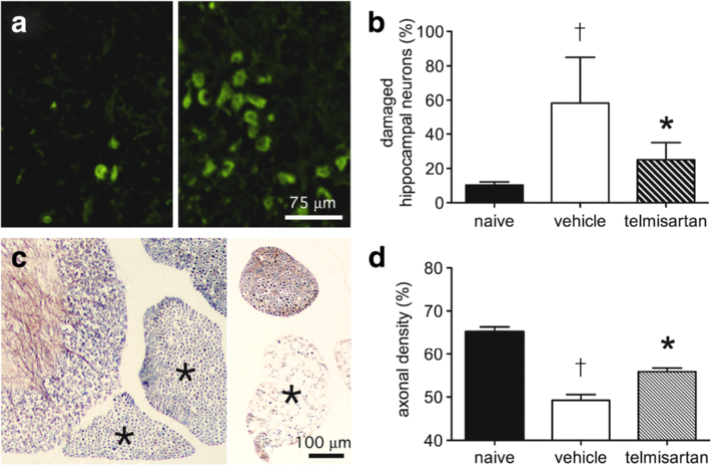
Telmisartan prevents irreversible neuronal damage and loss in the brains and spinal cords of NSV-infected mice. Fluoro-Jade C staining shows extensive labeling of hippocampal neurons in a mouse 7 days after NSV infection (*right panel*, **a**) compared to an uninfected control (*left panel*, **a**). *Bar* = 75 μm for both panels. Quantification of staining at this stage of infection as described in the “Methods” section shows that telmisartan (100 mg/kg/day) reduces damage to these neurons compared to animals treated with a vehicle control (**b**). In the lumbar spinal cord, silver staining shows prominent axonal loss in individual ventral nerve roots (each marked with an *) on day 7 post-infection (*right panel*, **c**) compared to an uninfected control (*left panel*, **c**). *Bar* = 100 μm for both panels. Quantification of axonal density in these ventral nerve roots as described in the “Methods” section shows that telmisartan treatment (100 mg/kg/day) reduces damage to lumbar motor neurons from which these axons arise compared to animals treated with a vehicle control (**d**). Student’s *t* test was used to analyze both the degree of cell damage in vehicle-treated versus naïve mice (†*p* < 0.0001 in both the hippocampus and spinal cord) as well as the degree to which drug treatment suppressed neuronal damage compared to those from vehicle-treated controls (*p = 0.0006 in the hippocampus; *p = 0.0005 in the spinal cord)

Based on this distribution of staining, the effects of telmisartan on the survival of hippocampal and lumbar ventral spinal motor neurons were quantified following NSV infection. Using Fluoro-Jade C labeling, an established histochemical marker of irreversible neuronal injury [46], damaged hippocampal neurons were easily identified on day 7 post-infection compared to naïve controls (Fig. 11a), and quantification of these cells showed that the drug exerted a measurable neuroprotective effect compared to vehicle-treated controls (Fig. 11b). Likewise, silver staining of sections through the lumbar spinal columns of these mice allowed the identification of lumbar ventral nerve roots carrying axons from lumbar ventral motor neurons (Fig. 11c); quantification of these axons showed that survival of these cells was also enhanced by telmisartan (Fig. 11d). Together, these findings demonstrate that a systemically administered ARB can penetrate the CNS to inhibit Nox activity and local ROS production, thereby limiting oxidative neuronal damage during NSV encephalomyelitis.

### Effects of Nox subunit deletion on telmisartan-mediated protection during NSV encephalomyelitis in vivo

**Fig. 12:**
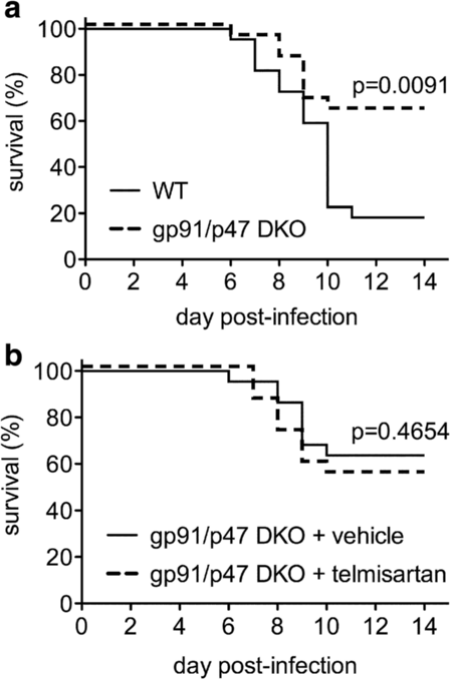
Nox-deficient mice are protected from NSV infection compared to WT controls, while telmisartan has no protective effect in Nox-deficient hosts. In the absence of any drug treatment, gp91/p47 DKO mice survived NSV infection much better than WT control animals (**a**). When gp91/p47 DKO mice were treated with either telmisartan (100 mg/kg/day) or a vehicle control, no survival difference between the two groups was observed (**b**). Statistical differences in outcome between these cohorts (*n* = 22 mice/group) were determined using a log-rank (Mantel-Cox) test. Individual *p* values are shown

Although multiple enzymes could theoretically be generating ROS in the CNS following NSV infection, Nox appears to be their primary source given the results of both our biochemical assays (Figs. 8c, d) as well as the absence of DHE staining in infected gp91/p47 DKO mice (data not shown). When gp91/p47 DKO mice were challenged with NSV, these hosts survived infection significantly better than WT controls (Fig. 12a). Furthermore, when telmisartan was given to cohorts of NSV-infected gp91/p47 DKO hosts, the survival benefit conferred by the drug was abolished compared to DKO animals treated with a vehicle control (Fig. 12b). These data confirm that Nox activity is pathogenic during NSV infection, and that Nox is the main therapeutic target of telmisartan in this alphavirus encephalitis model.

## Discussion

Although natural outbreaks of mosquito-borne encephalitis caused by alphaviruses remain rare events, aerosol transmissibility makes some of these pathogens potential bioterrorism agents [1]. Other dangerous features of alphaviruses include their potential to cause incapacitating disease, their relatively high infectivity for humans, the ease with which they can be mass produced, and the lack of effective countermeasures for disease prevention or control [1]. Studies undertaken in murine alphavirus encephalitis models have demonstrated that blocking host responses arising from activated microglial cells can protect infected hosts, often without suppressing CNS virus replication or spread [7, 10–14]. Although the molecular pathways leading to neuronal damage in infected mice are incompletely understood, strategies to subvert these injurious host responses remain fertile areas of investigation. Here, we implicate a novel injury mechanism in the CNS–ROS production via Nox activation—as an important contributor to alphavirus pathogenesis. We also demonstrate that an existing ARB capable of penetrating the BBB can effectively inhibit CNS Nox activity, prevent oxidative neuronal damage, and reduce disease morbidity and mortality in diseased animals without having any effect on local virus replication or clearance. This raises questions as to whether these drugs could have broader uses in the treatment of neurologic disorders where oxidative injury is known or hypothesized to occur.

Broadly defined, oxidative stress results from the increased production of both ROS and reactive nitrogen species (RNS) to an extent that cellular antioxidant defenses get overwhelmed. Both acute infections caused by herpes simplex virus and chronic infections caused by human immunodeficiency virus and measles virus are known to cause oxidative damage to neurons and glial cells within the CNS [21]. On the other hand, the extent to which the oxidative burst can benefit the infected host by limiting virus replication is unknown. Furthermore, ROS are known to participate in normal intracellular signaling, and Nox activation has now been shown to facilitate cellular Toll-like receptor (TLR) responses and augment type-1 interferon production following engagement of the cytoplasmic viral sensor, retinoic acid-inducible gene I (RIG-I) [47, 48]. Thus, local Nox activation could in theory either positively or negatively influence the outcome of CNS viral infection. We find that systemic administration of the ARB, telmisartan, potently suppresses Nox activity in both the brains and spinal cords of mice with NSV encephalomyelitis, effectively blocking local ROS production and preventing oxidative neuronal injury without altering virus replication or clearance. Evidence that the drug confers protection through a Nox-dependent pathway and not via some off-target effect comes from our observation that gp91/p47 DKO mice that do not generate any oxidative burst in the CNS following virus challenge, have improved disease outcomes compared to WT controls, and are completely unresponsive to telmisartan following NSV infection. Taken together, our data show that Nox activation is pathogenic in this disease setting and that this enzyme complex can be effectively targeted to benefit the host via systemic administration of a compound already widely used in humans.

While originally developed for use in treating hypertension and more recently shown to exert neuroprotective effects in stroke models [24–27], ARBs are now confirmed to have benefits during experimental neuroinflammation. In experimental autoimmune encephalomyelitis (EAE), the principal mouse model of multiple sclerosis (MS), systemically administered ARBs can suppress disease severity [49–51]. One study showed that ARB treatment disrupted local astrocyte and microglial production of the inflammatory factor, transforming growth factor-beta (TGF-β), and suppressed myeloid cell recruitment to the CNS [51]. The authors of this study inferred that actions on these glial cells were primary and that suppressed inflammatory cell infiltration into diseased CNS tissue was a secondary effect [51]. In a mouse model of traumatic brain injury, a single dose of telmisartan given 6 h after injury caused reduced perilesional staining for CD68, ionized calcium binding adaptor molecule 1 (Iba-1), and myeloperoxidase (MPO) at 72 h, while a similar treatment 24 h after injury had no such effect [52]. In this setting, however, the cells targeted by telmisartan were not specifically identified, and its effects proved largely dependent on peroxisome proliferator-activated receptor gamma (PPARγ) agonism as opposed to AT1R blockade [52]. Other in vitro studies have also shown that the anti-inflammatory actions of ARBs on myeloid cells correlate with their PPARγ agonist potency [53]. Although our work does not specifically address the role of PPARγ in NSV pathogenesis, we found no evidence that telmisartan conferred benefit in the setting of Nox deficiency or exerted a more global anti-inflammatory effect. This variability in response might be explained by its capacity to act on multiple targets in different disease models. Indeed, in some settings telmisartan may directly enter target cells and scavenge intracellular ROS fully independent of its AT1R blocking effects [54].

The renin-angiotensin system (RAS) has now been recognized to exert direct actions within the CNS and to serve roles beyond the neural control of cardiovascular function [55, 56]. These observations have spawned many studies to examine the cellular localization of RAS components within the brain. There is some consensus, for example, that AGT and the cleaving enzymes needed for Ang II synthesis exist in distinct neural cell types [55]; this would imply that a network of cells contributes to the final production of this mediator. As for Ang II receptors, most reports validate that both AT1R and AT2R are found at varying levels throughout the brain, although debate still rages surrounding their relative localization to neurons versus glial cells [55, 56]. While some genetic models suggest that Ang II receptors are found primarily on neurons under steady state conditions [57, 58], co-localization using glial-specific reporter mice has not been described until now. Furthermore, AT1R are found on peripheral immune cells [59] and are abundant on CNS-infiltrating myeloid cells during both EAE and MS [50]. Using CX3CR1^GFP/+^ mice that label both microglia and peripheral monocytes [39], histochemical staining of tissue sections as well as direct physical separation of CNS myeloid cell subsets by flow cytometry allowed us to confirm that AT1R localizes principally to CX3CR1+ and to CD45+/CD11b+ cells during NSV infection. Ongoing studies will use WT and AT1R KO mice to create bone marrow chimeras in order to confirm that hematopoietic cells are the main target of telmisartan in this disease, with a longer-term goal being the creation of a conditional AT1R KO mouse. Nonetheless, our current findings fit with other studies showing that aberrant host responses arising from activated myeloid cells contribute to NSV pathogenesis [7, 10, 13], even if they do not yet shed any light on the source or mechanism of local Ang II production following NSV infection.

Finally, it bears considering that another group recently studied the role of Ang II and ARB signaling in a closely related infection model caused by another alphavirus, Venezuelan equine encephalitis virus (VEEV), in rats. These investigators found that Ang II expression increased rapidly in the CNS following VEEV infection, but that daily treatment of animals with losartan (30 mg/kg) actually accelerated death compared to rats given a vehicle control [60]. In their hands, losartan suppressed VEEV-mediated induction of the pro-inflammatory mediators, IL-1α and CCL2, as well as the anti-inflammatory compound, IL-10, but had no effect on IL-6 levels in the brain [60]. Drug treatment also reduced vascular pathology in the CNS of VEEV-infected animals, but had no quantitative effects on cellular infiltration into the brain. They concluded that Ang II-driven neuroinflammation could be a host defense mechanism that limits virus damage and favors survival [60]. Unfortunately, however, their conclusions contradict numerous other studies showing that host responses actually drive VEEV pathogenesis and directly contribute to mortality [61–63]. We otherwise remain unable to explain why our results differ so dramatically from these investigators’ conclusions using a related encephalitis model.

## Conclusions

Our data show that components of the RAS are induced in the CNS during the early stages of alphavirus encephalitis, and that the ARB, telmisartan, acts in a highly targeted manner to suppress Nox activity in both endogenous and CNS-infiltrating myeloid cells and protect infected hosts from lethal disease. Impaired ROS production reduces neuronal injury, and the abrogation of clinical protection in Nox-deficient hosts confirms that these enzymes are the main target of this drug in this setting. Given widespread use in humans, this ARB may also confer benefit in treating neurologic disorders where oxidative injury is known or hypothesized to occur.

## Additional files

Additional file 1: Figure S1. Representative Western blots show induction of AT1R in both the brain and spinal cord over the course of acute NSV infection relative to the expression of a β-actin loading control in each tissue sample (TIF 352 KB). (TIF 297 kb) Additional file 2: Figure S2. Representative Western blots show induction of the Nox subunits, gp91 and p47, in both the brain and spinal cord over the course of acute NSV infection relative to the expression of a β-actin loading control in each tissue sample (TIF 492 KB). (TIF 424 kb)

## Abbreviations

4-HNE: 4-Hydroxynonenal
ACE: Angiotensin converting enzyme
AGT: Angiotensinogen
Ang II: Angiotensin II
ANOVA: Analysis of variance
ARB: Angiotensin II receptor blocker
AT1R: Type-1 angiotensin II receptors
AT2R: Type-2 angiotensin II receptors
BBB: Blood-brain barrier
BSL3: Biosafety level 3
CNS: Central nervous system
cDNA: complementary DNA
CX3CR1: CX3C chemokine receptor-1
DHE: Dihydroethidium
DKO: Double knockout
DMEM: Dulbecco’s modified Eagle’s medium
EAE: Experimental autoimmune encephalomyelitis
EIA: Enzyme immunoassay
FBS: Fetal bovine serum
GFP: Green fluorescent protein
HBSS: Hank’s balanced salt solution
HRP: Horseradish peroxidase
i.p.: Intraperitoneal
KO: Knockout
MFI: Mean fluorescence intensity
MNC: Mononuclear cells
MPO: Myeloperoxidase
MS: Multiple sclerosis
NADPH: Nicotinamide adenine dinucleotide phosphate
NGS: Normal goat serum
Nox: NADPH oxidase
NSV: Neuroadapted Sindbis virus
OD: Optical density
PBS: Phosphate-buffered saline
PFA: Paraformaldehyde
pfu: plaque forming units
PPARγ: Peroxisome proliferator-activated receptor gamma
qPCR: Quantitative PCR
RAS: Renin-angiotensin system
RIG-I: Retinoic acid-inducible gene I
RNS: Reactive nitrogen species
ROS: Reactive oxygen species
SDS: Sodium dodecyl sulfate
SEM: Standard error of the mean
SOD: Superoxide dismutase
TBS: Tris-buffered saline
TGF-β: Transforming growth factor-beta
TLR: Toll-like receptor
VEEV: Venezuelan equine encephalitis virus
WEEV: Western equine encephalitis virus
WT: Wild type
