# Supplementary figures and images for: Type-1 angiotensin receptor signaling in central nervous system myeloid cells is pathogenic during fatal alphavirus encephalitis in mice
Source: J Neuroinflammation. 2016 Aug 25;13(1):196. doi: 10.1186/s12974-016-0683-7 (PMC5000512; doi:10.1186/s12974-016-0683-7)

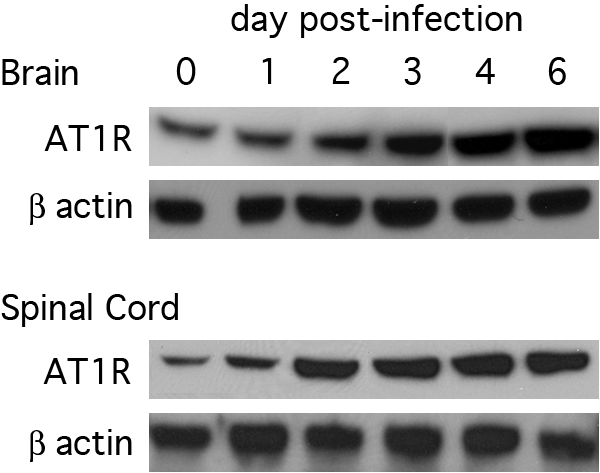

Supplement: Additional file 1: Figure S1. — Representative Western blots show induction of AT1R in both the brain and spinal cord over the course of acute NSV infection relative to the expression of a β-actin loading control in each tissue sample (TIF 352 KB). (TIF 297 kb) [file 12974_2016_683_MOESM1_ESM.tif]

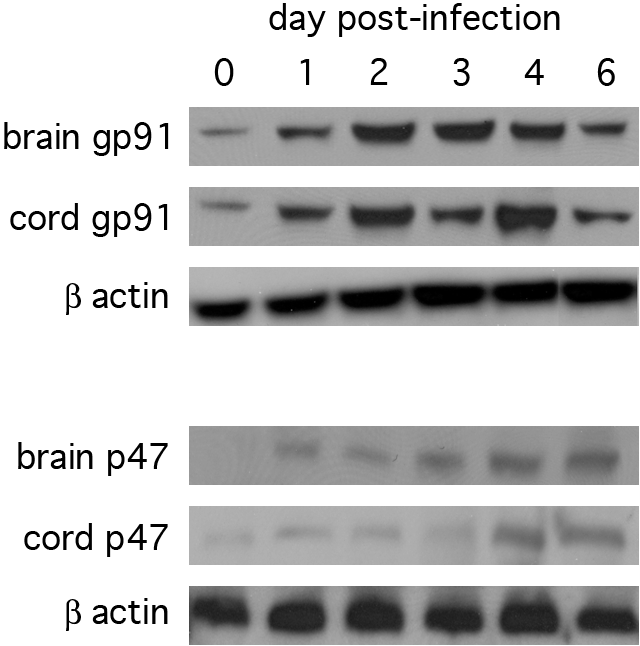

Supplement: Additional file 2: Figure S2. — Representative Western blots show induction of the Nox subunits, gp91 and p47, in both the brain and spinal cord over the course of acute NSV infection relative to the expression of a β-actin loading control in each tissue sample (TIF 492 KB). (TIF 424 kb) [file 12974_2016_683_MOESM2_ESM.tif]
